# Partial Replacement of Soyabean Meal with Defatted Black Soldier Fly (*Hermetia illucens* L.) Larvae Meal Influences Blood Biochemistry and Modulate Oxidative Stress, but Not Growth Performance of Pigs

**DOI:** 10.3390/ani15081077

**Published:** 2025-04-08

**Authors:** Gergana Yordanova, Radka Dimitrova Nedeva, Apostol Petrov Apostolov, Stephen Charles Mansbridge, Isobel Margaret Whiting, Alexander Mackay Mackenzie, Galina Dimitrova Nikolova, Yanka Dimitrova Karamalakova, Vasil Radoslavov Pirgozliev

**Affiliations:** 1 Agricultural Academy, Agricultural Institute, 9700 Shumen, Bulgaria; gerganaarshspb@abv.bg (G.Y.); r.nedeva@abv.bg (R.D.N.); apostolov_a@abv.bg (A.P.A.); 2 Animal Science Research Centre, Agriculture and Environment Department, Harper Adams University, Newport, Shropshire TF10 8NB, UK; smansbridge@harper-adams.ac.uk (S.C.M.); iwhiting@harper-adams.ac.uk (I.M.W.); amackenzie@harper-adams.ac.uk (A.M.M.); 3 Social Medicine, Health Management and Disaster Medicine, Disaster Medicine, Medical Faculty, Trakia University, 11 Armeiska Str., 6000 Stara Zagora, Bulgaria; galina.nikolova@trakia-uni.bg; 4 Department of Medical Chemistry and Biochemistry, Medical Faculty, Trakia University, 11 Armeiska Str., 6000 Stara Zagora, Bulgaria; yanka.karamalakova@trakia-uni.bg

**Keywords:** insect meal, black soldier fly, growing pigs, growth performance, digestible energy, nutrient digestibility, blood biochemistry, oxidative stress

## Abstract

The production of soybean meal (SBM) can be linked to various issues related to the environment (e.g., deforestation, water waste, and transportation costs), and reducing its inclusion in pig diets by using alternative protein sources, such as insect meal, is an important challenge for nutritionists. This study aimed to compare the productive performance, dietary digestible energy (DE), nutrient digestibility, and some blood indices of growing Danube White pigs fed graded levels of Black Soldier fly (*Hermetia illucens* L.) larvae meal (BSFLM) at 0, 30, 60, 90, and 120 g/kg of diets, in replacement of SBM for 38 days, from 119 to 157 d old. Each diet was fed to eight pigs in individual boxes following randomization. Pigs grew according to breeders’ recommendations and did not have any clinical health problems. Replacing SBM did not change (*p* > 0.05) the pigs’ growth performance and DE, as only dietary fat digestibility increased in a linear fashion (*p* < 0.001), possibly due to the high BSFLM, i.e., the high-fat inclusion rate. There was a simultaneous rise in some oxidative damage indicators and an increase in antioxidant status, thus suggesting that further research involving longer feeding periods is needed to identify a potential time sequence of events. Overall, BSFLM is a promising ingredient in pig nutrition.

## Simple Summary

Exploring alternative protein sources to soybean meal helps minimise the environmental impact of pig production. The objective of the present study was to assess the impact of replacing 120 g/kg of dietary soybean meal with insect meal on growth performance, energy and nutrient digestibility, and inflammatory-mediated oxidative damage. Replacing over half of dietary soybean meal with insect meal did not impact the growth performance and energy and nutrient digestibility of the pigs. However, Black Soldier fly meal did promote some inflammatory pathways; however, this did not impact performance. Incorporating insect meal into dietary formulations could be a valuable strategy for enhancing the sustainability of commercial pig production.

## 1. Introduction

Pork is the second most consumed meat worldwide after poultry, and pig production is one of the fastest growing livestock sectors globally [1,2,3]. To meet the growing global demand for pig meat, the net production of pork worldwide in 2024 reached 115.56 million metric tons carcass weight, i.e., 6% more compared to 108.85 million metric tons in 2013 [3]. It is widely accepted that feed comprises about 60–70% of the total costs in intensive pig production, particularly in Europe, as protein is the most expensive dietary component [4]. The increased production of pork will inevitably increase the demand for ingredients to formulate diets that include protein sources for pigs. Globally, soybean meal (SBM) is the main protein source used in non-ruminant diets. However, due to an increase in the global demand, SBM is increasingly unavailable and expensive for pig producers. Soybean meal production can also be associated with deforestation, negatively impacting the environment and driving climate change [5,6,7]. Considering the growing demand for high-quality protein sources, the potential negative environmental impact of producing these sources, and the constant increase in net pork production [5,8], reliance on SBM is not maintainable [6]. Therefore, the modern pig industry requires sustainable, easily produced alternative protein sources to supplement or replace SBM in feed. Insect larvae meal is rich in protein, fat, and calcium and is often regarded as a promising protein source for developing more sustainable pig production systems [9,10,11,12]. It has also been reported that the nutritional profile of larvae meal from Black Soldier fly (*Hermetia illucens*; BSFLM) [13] and the digestibility of amino acids [14] closely resembles that of SBM and can be used as a potential SBM alternative in broiler diets. In addition, insect meal and insect fat are good sources of available energy for non-ruminant animals [13,15,16,17,18]. Insects can contribute to a circular economy by growing on organic byproducts, reducing competition with humans for resources [19,20,21].

BSFLM has been used at the expense of SBM as an ingredient in diets for fish [22,23,24], poultry [13,25,26], and pigs [1,27,28,29,30] with variable success. However, most of the research on feeding BSFLM to pigs has been focused on studies with young pigs over short feeding periods. Newton et al. [27] reported that a 33% feed inclusion of BSFLM increased feed intake but reduced dry matter digestibility in 5-week-old barrows. In later research, Newton et al. [28] found that the replacement of dried plasma increased the growth performance of early weaned pigs in 50% dietary BSFLM but reduced it in 100% dietary BSFLM. When replacing 50 g/kg (30%) and 100 g/kg (60%) of dietary SBM with BSFLM in diets of weaned pigs, Biasato et al. [30] reported no overall differences in growth performance, nutrient digestibility, or gut morphology when feeding BSFLM. Spranghers et al. [29] did not find differences in the growth performance of weaning pigs when replacing 8% of SBM with BSFLM, but the ileal protein digestibility of the BSFLM diet was lower. Chia et al. [1] managed to completely replace fish meal with BSFLM in pig diets for 59 days from 19.6 to 53.4 kg body weight. However, the diets fed by Chia et al. [1] did not contain any SBM, and the BSFLM only represented 18.5% of the diet. By feeding 120 g/kg of full-fat Black Soldier fly larvae, Boontiam et al. [31] reported greater nutrient digestibility, duodenal villus morphometry, and overall intestinal health in post-weaning pigs. Thus, it is still unclear whether BSFLM can completely replace SBM, and there is limited information on the influence of BSFLM on the health response of growing pigs.

The status of haematological and biochemical variables depends on several factors, including the diet [32]. Thus, analysing the blood profile of pigs fed BSFLM, a novel protein source, can provide useful information in animal production, health, and welfare [32,33,34]. There is evidence from multiple studies that BSFLM is associated with haematological changes. For example, feeding dietary BSFLM tended to linearly increase the peripheral blood lymphocytes and neutrophils in growing pigs [30]. Chia et al. [1] reported lower platelet counts in pigs fed 90, 145, and 185 g/kg of BSFLM compared to pigs fed 0 and 120 g/kg of BSFLM. The same authors found an increase in neutrophils and a tendency of a reduced count of lymphocytes when 185 g/kg of BSFLM was fed. Research by Boontiam et al. [31] found that 120 g/kg of full-fat Black Soldier fly larvae in weaned pigs diets improved blood immunoglobulin A and glutathione peroxidase and decreased tumour necrosis factor-alpha levels. When feeding 150 g/kg of protein hydrolysate from BSFL, Li et al. [35] observed a positive effect on palatability and enhanced antioxidant, anti-inflammatory, and immune capacity in cats. However, comprehensive confirmation of the effects of dietary BSFLM on growing pig haematology is required.

The main objectives of this study were to evaluate the effect of partially substituting SBM with BSFLM on (a) growth performance variables, (b) dietary digestible energy (DE) and nutrient digestibility coefficients, and (c) haematological, biochemical, and blood plasma antioxidant and oxidative stress indices in growing Danube White pigs between 30 and 60 kg live weight.

## 2. Materials and Methods

### 2.1. Insect Meal and Experimental Diets

For this experiment, a BSFLM sample was obtained from Hexafly^TM^ (Navan, Co., Meath, Ireland; Table 1). The BSFLM was from larvae no older than 17 days, and the production process followed EC regulation EU 142/2011 [36], as explained previously [13].

Five diets in total (Table 2) were formulated in mash form similar to growing pig nutrient recommendations (https://ahdb.org.uk/knowledge-library/nutrition-guidance-for-pigs, accessed on 1 April 2025), as the DE was slightly greater due to breed differences (Agricultural Institute Shumen, 9700, Bulgaria). All diets were based on maize, wheat, barley, and SBM and contained graded levels of BSFLM, primarily at the expense of SBM; the control diet did not contain BSFLM (T0); the diet contained 30 g/kg of BSFLM (T30); the diet contained 60 g/kg of BSFLM (T60); the diet contained 90 g/kg of BSFLM (T90); the diet contained 120 g/kg of BSFLM (T120). Diatomaceous earth (Multi-Mite, Wiltshire, UK) was used as an acid insoluble ash (AIA) digestibility marker and was included at 20 g/kg on the top of each diet.

### 2.2. Animals, Experimental Design, and Sample Collection

The 38-day feeding bioassay was approved by the Agricultural Academy Research Ethics Committee (Project number Ж182, Agricultural Academy, Sofia). The study was conducted at the pig research facility of the Agricultural Institute (Shumen, 9700, Bulgaria), and all experimental procedures described complied with the European and Bulgarian legislation regarding the protection of animals used for experimental and other scientific purposes [37]. The manuscript was prepared in compliance with the ARRIVE 2.0 guidelines [38].

A total of 40 male castrates and female Danube White pigs were selected for the experiment and allocated to 40 individual pens equipped with an individual feeder and drinker, each with a 2 m^2^ floor area. The ambient temperature was maintained at 18–20 °C, and the relative humidity was 65–71%. The experiment started when pigs were 119 d old, and the mean body weight was 31.86 kg (SDEV ± 0.089). Each diet was fed to 8 pens following randomisation. The study continued for 38 days, and pigs were weighed at the beginning, at 119 d old, and at the end of the bioassay, at 157 d old, inclusively. Pigs were fed ad libitum, receiving two portions of feed daily. Before the morning feeding, all feed residuals were collected and weighed to determine daily feed intake (FI). During the last 4 days of the study, the faeces were collected following standard methodology [39,40].

After the collection, faeces were oven-dried at 60 °C, milled to pass through a 0.75 mm sieve, and further analysed for acid insoluble ash (AIA) as an indigestible marker, dry matter (DM), nitrogen (N), ether extract (EE), and gross energy (GE). Daily FI, daily weight gain (WG), and the feed conversion ratio (FCR) were also determined.

During the last day of study, blood samples were obtained before the morning feeding from the orbital venous sinus of each pig by employing a closed system method [41]. Samples for plasma analyses were collected into lithium heparin tubes (Vacusera, Izmir, Turkey), centrifuged, plasma aliquoted into Eppendorf tubes, and stored at −80 °C prior to subsequent analyses. Whole blood samples were collected in EDTA tubes (Vacusera, Izmir, Turkey) and stored at room temperature for haematological analysis within 6 h of sampling.

### 2.3. Laboratory Analyses

The analyses of feed, BSFLM, and faeces were performed at the Agricultural Institute (Shumen, Bulgaria) and the Elizabeth Creek Laboratories (Harper Adams University, Shropshire, UK). The analyses of blood were completed at the Agricultural Institute (Shumen, Bulgaria) and the Medical Faculty, Trakia University (Stara Zagora, Bulgaria).

#### 2.3.1. Feed, BSFLM, and Faeces

The sample preparation of feed and faeces were performed following standard procedures as described elsewhere [42]. Minerals in BSFLM and diets were determined following the method described by Tanner et al. [43]. The DM, N, and fat (as ether extract; EE) in samples were determined, with values of 934.01, 968.06, and 991.36 [44], respectively. Crude protein (CP) in faeces and standard dietary components was calculated as N × 6.25, although CP in BSFLM was calculated as N × 5.60 (Table 1). The calculated CP in BSFLM was used for dietary formulation. The GE in samples was determined with an isoperibol bomb calorimeter (Model 6200, Parr Instrument Co., Moline, IL, USA) as described previously [45]. Amino acids (AAs) in BSFLM and diets were analysed as described elsewhere [46]. The AIA in feed and faeces was determined following the method by Van Keulen and Young [47]. Total fibres (TFs), acid detergent fibre (ADF), and acid detergent lignin (ADL) in BSFLM and diets were determined following the standard procedure [48]. The content of chitin in BSFLM was determined as previously described [13,49].

#### 2.3.2. Haematological Variables

The whole blood samples were analysed for red blood cells (RBCs), white blood cells (WBCs), platelets (Plts), haematocrit (Hct), and haemoglobin (Hb) as described previously [40].

#### 2.3.3. Antioxidant and Oxidative Damage Indicators

Blood plasma samples were analysed for antioxidant indicators and lipid peroxidation, including superoxide dismutase (SOD) activity, catalase (CAT) activity, glutathione peroxidase (GPx) activity, total antioxidant capacity (TAC), and malondialdehyde (MDA) content, using ELISA kits (Wuhan Fine Biotech Co., Ltd., Wuhan, China), as described by Georgiev et al. [50].

Oxidative damage indicators, including reactive oxygen species (ROS), nitric oxide radicals (•NO), ascorbate radicals (•Asc), nitroxide protein oxidation (5-MSL), and membrane-permeable piperidine nitroxide (•O_2_^−^/TEMPOL) were determined with electron paramagnetic resonance (EPR) spectroscopy analyses, following standard methodologies [51,52,53,54,55,56]. All EPR analyses were performed with five-fold measurements in recorded EPR spectra with the following characteristics: a 3503–3515 G centre field; 6.42–20.00 mW microwave power; and 5–10 G modulation per sample. The results are presented in arbitrary units (a.u.). EPR processing was performed using the WIN-EPR *SimFonia* 1.2/6130860 software.

#### 2.3.4. Products of Protein and Lipid Oxidation

The determination of 8-hydroxydeoxyguanosine (8-OhdG), protein carbonyl (PC) damage, and advance glycation end products (AGEs) were determined via enzyme-linked immunosorbent assay (ELISA) kits (Wuhan Fine Biotech Co., Ltd., Wuhan, China), following the manufacturer’s instructions.

#### 2.3.5. Inflammatory Markers

According to the manufacturer’s instructions, proinflammatory cytokines, including interleukins (IL-1β, IL-6, and IL-10), tumour necrosis factor-α (TNF-α), and interferon-gamma (INF-γ), were analysed using enzyme-linked immunosorbent assay (ELISA) kits (Wuhan Fine Biotech Co., Ltd., Wuhan, China), as previously described [56].

### 2.4. Calculations

Dietary DE values of the experimental diets were determined following the indigestible marker technique, as described elsewhere [39]:

$$DE=GE\;Diet-\frac{\left(GE\;Faeces\times AIA\;Diet\right)}{AIA\;Faeces}$$

where GE is the gross energy in the diet or faecal samples and AIA is the acid insoluble ash in the diet or faecal samples.

The total tract nutrient digestibility (ND) coefficients were calculated using the following equation [40]:

$$ND=\frac{\left(N/AIA\right)\;Diet\;–\left(N/AIA\right)\;Faeces}{(N/AIA)\;Diet}$$

where (N/AIA) Diet is the ratio of the respective nutrient to acid insoluble ash in the diet and (N/AIA) Faeces is the ratio of the respective nutrient to acid insoluble ash in faecal samples.

### 2.5. Statistical Analysis

Statistical analyses were performed using the GenStat (23rd edition) statistical software (IACR Rothamsted, Hertfordshire, UK). The data were analysed using ANOVA. Orthogonal polynomial contrasts were used to compare treatment differences for linear and quadratic relationships with increasing levels of BSFLM. Data were checked for homogeneity and normality of residuals prior to ANOVA. Results were considered significant at *p* < 0.05. Data are expressed as means and their pooled standard errors of means (SEMs).

## 3. Results

All animals completed the study in a good health with no mortalities.

### 3.1. Insect Meal

Table 1 presents information regarding the analysed chemical composition of the BSFLM used in this study. The DM content of BSFLM was 973 g/kg, and the GE was 21.15 MJ/kg. The content of CP (N × 5.6) was high, at 454 g/kg, followed by crude fat, at 171 g/kg, and ash, at 122 g/kg. The meal contained 79.5 g/kg of ADF, 20.4 g/kg of ADL, and 59.1 g/kg of chitin. Calcium was the main mineral, at 39.7 g/kg, in the BSFLM, followed by P, at 11.9 g/kg, and S, at 4.3 g/kg. Leucine and lysine were the main indispensable AAs, although aspartic and glutamic acids were the main dispensable AAs. Tryptophane was the least presented indispensable AA, and cystine was the lowest dispensable AA. The determined energy, proximate analyses, Ca, P, fibre, and AA contents of the experimental diets are presented in Table 3. The control diet (T0) had slightly lower CP and crude fat but greater overall AA contents compared to the other diets. The diet containing 90 g/kg of BSFLM had lower lysine and total AA contents. Dietary crude fat gradually increased with the BSFLM inclusion rates.

### 3.2. Growth Performance, Digestible Energy, and Nutrient Digestibility

The start and the end body weights of the pigs were 31.86 kg (SDEV ± 0.089) and 59.66 kg (SDEV ± 0.912), respectively (Table 4). There were no significant differences (*p* > 0.05) within the studied productive performance variables. Information about dietary DE and nutrient digestibility coefficients is presented in Table 4. Fat digestibility was the only variable to significantly differ, whereby the control diet had the lowest and the diet containing 120 g/kg of BSFLM had the highest digestibility coefficient (*p* = 0.002), which followed a linear response (L < 0.001).

### 3.3. Haematological Variables

Haematological analyses are presented in Table 5. The only difference was found in WBC, as the pigs fed the diet containing 120 g/kg of BSFLM had a greater value compared to the rest (*p* = 0.016), following a curvilinear pattern (L = 0.034; Q = 0.005).

### 3.4. Antioxidant and Oxidative Damage Indicators

Table 5 contains information regarding the antioxidant and oxidative damage indicators of pig blood plasma. The SOD activity was lower (*p* < 0.001) in the blood plasma of pigs fed the control diet, but this was a complex response across levels (DEV < 0.001) regarding dietary BSFLM. The CAT activity was greater (*p* = 0.002) in pigs fed the T0 and T30 diets, decreasing linearly (L < 0.001) with the increase in dietary BSFLM. The GPx activity was greater (*p* < 0.001) in pigs fed the control diet, and similar to the SOD activity, there was no simple pattern (DEV < 0.001). Similarly, the TAC activity was lower (*p* < 0.001) in the control-fed pigs (*p* < 0.001), following a curvilinear pattern (L < 0.001; Q = 0.007). The MDA levels and mean lipid peroxidation were lower in the control-fed pigs (*p* < 0.001) and linearly increased (L < 0.001) with the increase in BSFLM in diets.

The production of ROS was lower (*p* < 0.001) in the control-fed pigs, without a clear pattern of the BSFLM-level response (DEV < 0.001). Similar to TNF-α, the response of the •NO concentration was lower in the T0 and T120 diets (*p* < 0.001), and there was a quadratic response to the dietary level of BSFLM (Q < 0.001). The concentration of •Asc was lower (*p* < 0.001) in the plasma of pigs fed the T0 diet and linearly increased (L < 0.001) with dietary BSFLM levels. The concentration of 5-MSL (-SH conformation in albumin; protein oxidation) was significantly elevated (*p* < 0.001) with all levels of dietary BSFLM inclusion. The response of TEMPOL (•O_2_^−^ radical concentration) was similar to those of PC (*p* < 0.001; L < 0.001; Q < 0.001).

### 3.5. Products of Protein and Lipid Oxidation

Pigs fed the T60 diet had the highest 8-OhdG concentration compared to the others (*p* = 0.004), and the response to the dietary level of BSFLM was quadratic (Q = 0.004; Table 5).

Pigs fed the T0 diet had the lowest PC concentration (*p* < 0.001), and the response to the BSFLM level was curvilinear (L < 0.001; Q < 0.001). The AGEs were greater in pigs fed the T0 diet (*p* = 0.011), but the response to the dietary BSFLM level was clear but complex (DEV = 0.034).

### 3.6. Inflammatory Markers (Cytokines)

IL-1β (*p* = 0.002) and IL-6 (*p* < 0.001) were lower in pigs fed the T0 diet, and both followed a curvilinear pattern (L < 0.05; Q = 0.05) (Table 5). IL-10 had a higher concentration (*p* < 0.001) in T0-fed pigs, although there was not a clear pattern following the dietary BSFLM level (DEV = 0.004). The concentration of INF-γ was lower (*p* = 0.002) in the plasma of pigs fed the control diet and linearly increased (L < 0.001) with dietary BSFLM levels. The TNF-α concentration was lower in pigs fed the control and 12% insect meal diets (*p* < 0.001), and there was a quadratic response to the dietary BSFLM level (Q < 0.001).

## 4. Discussion

The chemical composition of BSFLM was within the expected range [14,21,57]. Although we obtained the BSFLM from the same producer, there were some small differences with the previous batch [13]. It is well recognised that there are variations in the fat, protein, and chitin content of BSFLM, which usually relate to differences in larvae age and the rearing substrate [9,58], thus explaining the observed inconsistencies. The CP values differed primarily due to the use of a different conversion factor, i.e., 5.6 in the present batch vs. 6.25 in the previous BSFL batch [13].

The overall composition of the experimental diets was within the expected range. Minor differences between the analysed and calculated values of the experimental diets may be attributed to differences in the ingredients and values used in the formulation software.

The animals grew as expected according to common breeding standards (Agricultural Institute, Shumen, Bulgaria), in agreement with previous studies [41]. The lack of difference in any of the growth performance variables showed that replacing 120 g/kg (over 50%) of the SBM with BSFLM in practical dietary formulations for growing pigs is possible. This is in accord with previous research [29,30] where up to 100 g/kg of BSFLM inclusion levels in replacement of SBM did not result in a significant difference in growth performance. Boontiam et al. [31] also reported that the addition of 120 g/kg of dietary Black Soldier fly larvae did not change growth performance but improved the gut health of weaning pigs reared in low hygienic conditions. Chia et al. [1] showed that growing pigs can tolerate diets containing 185 g/kg of BSFLM in replacement of fish meal in SBM-free diets, further confirming the suitability of BSFLM in pig nutrition. Studies with fish [59] also showed the potential of BSFLM as a dietary protein ingredient. However, research with broilers [13,60,61] showed that 30% and 50% replacement of SBM with BSFLM may lead to reduced growth performances. Chobanova et al. [13] suggested that this may be due to inaccuracies in dietary formulation assumptions regarding non-protein nitrogen (NPN) in BSFLM, AA imbalances, and relatively high chitin content. In the present study, dietary NPN and the AA balance were considered and adjusted appropriately. In addition, growing pigs have a more developed gastrointestinal tract, and nutrient availability may not be affected as much by chitin, which is confirmed by the results regarding DE and nutrient digestibility in this study.

Generally, the haematology variables agree with previous reports on growing pigs [1,30,40] and were within the expected range reported for pigs [62], indicating that the animals status of health was good. Similar to previous reports [1,30], in the present study, the count of overall WBCs slightly increased with the increased dietary BSFLM. Neutrophils, which represent over 50% of the WBCs, are involved in immune responses through microbial sterilisation and macrophage attraction [63]. The fat from Black Soldier fly larvae contains medium-chain saturated fatty acids, e.g., lauric acid [18], with pronounced antimicrobial properties [29,64]. Ahlante et al. [65] reported increased leucocytes and neutrophil counts in rabbits fed diets containing coconut oil, i.e., a product rich in medium-chain saturated fatty acids. Thus, the increase in WBCs in our study may indicate neutrophil activation in pigs fed high BSFLM levels. A review by Jozefiak and Engberg [66] further emphasises the antimicrobial properties of insect meal, showing that in addition to the supply of dietary protein and fat, insects potentially bring nutraceutical benefits. However, in our study, there appears to be an activation of the innate immune system, which was previously identified by Fariz Zahir Ali et al. [67], thus suggesting that chitin or bacterial proteins originating from the Black Soldier fly gut microbiome (frass) may be a potential reason for the observed responses. The overall status of the pigs fed BSFLM showed increased indications of inflammatory stimulation. This manifests as increased levels of IL-1β, Il-6, and TNF-α (without T120) along with increased values for ROS, •NO, and TNF-α were reduced when T120 was fed. This contrasts with the data on BSFLM-fed cats, which showed a decrease in proinflammatory cytokine levels [35]. However, a consistent level of fat was formulated across all cat diets [68], but in our study, the T90 and T120 diets contained more fat originating from the BSFLM than T0, T30, and T60. Increases in dietary fat intake have been shown to increase inflammatory signalling pathways associated with the transcription factor NF-kB that regulates various aspects of the variables indicated above [69]. Additionally, IL-10 levels in the T0-fed pigs were significantly lower compared to those fed with BSFLM. IL-10 is a known inhibitor of NF-kB signalling.

In response to the proinflammatory cytokines, ROS, PC, and TEMPOL increase; BSFLM-fed pigs had greater activity of the antioxidant SOD enzyme, •Asc, and TAC. However, the observed contradiction resulted in the simultaneous increase in some oxidative damage indicators (MDA, ROS, NO, and 5-MLS), and increases in the antioxidant status suggests a homeostatic response. Further research is therefore needed to identify a potential time sequence of events, i.e., which occurs first, the oxidative damage or the protection. Thus, a longer feeding period with a sequential sample collection is recommended to allow for a better understanding of the impact of BSFLM on immune-mediated responses in pigs.

## 5. Conclusions

Replacing over half of dietary SBM with BSFLM, 120 g/kg in this study, in practical diets did not change dietary DE, nutrient digestibility, or the productive performance of growing pigs. Overall, feeding BSFLM increased lipid oxidation and proinflammatory blood plasma biomarkers and improved the antioxidative status of the animals. Further research involving longer feeding periods may allow for a better understanding of the long-term suitability of feeding BSFLM for pig production.

## Figures and Tables

**Table 1:** Proximate, carbohydrate, mineral, and amino acid composition of Black Soldier fly larvae meal (as fed basis) ^a^.

Proximate and Carbohydrate Composition (g/kg)		Indispensable Amino Acids (g/kg)	
Dry matter	972.7	Arginine	21.36
Gross energy (MJ/kg)	21.15	Histidine	17.21
Crude protein (N × 5.60)	454	Isoleucine	20.43
Crude fat	171	Leucine	33.31
Ash	122	Lysine	32.61
Acid detergent fibre	79.5	Methionine	9.08
Acid detergent lignin	20.4	Phenylalanine	20.75
Chitin	59.1	Threonine	18.92
Minerals		Valine	29.21
Boron (mg/kg)	3.4	Tryptophane	7.68
Calcium (g/kg)	39.7	Dispensable amino acids (g/kg)	
Cobalt (mg/kg)	0.09	Alanine	30.38
Copper (mg/kg)	23.9	Aspartic acid	47.41
Magnesium (g/kg)	4.0	Cystine	3.32
Manganese (mg/kg)	132.3	Glycine	25.09
Molybdenum (mg/kg)	0.9	Glutamic acid	46.89
Phosphorus (g/kg)	11.9	Proline	24.93
Potassium (g/kg)	13.5	Serine	19.54
Selenium (mg/kg)	0.3	Tyrosine	30.36
Sodium (mg/kg)	77.0		
Sulphur (g/kg)	4.3		
Zinc (mg/kg)	120.7		

^a^ Analysed in technical duplicates.

**Table 2:** Composition of pig diets (g/kg, as fed) used in the experiment.

Ingredients (g/kg)/Diets	T0	T30	T60	T90	T120
Maize	200.0	200.0	200.0	200.0	200.0
Wheat	347.8	349.1	351.7	354.2	356.5
Barley	200.0	200.0	200.0	200.0	200.0
Soybean meal	228.0	199.0	169.0	139.0	109.0
BSFLM ^a^	0.0	30.0	60.0	90.0	120.0
Lysine	1.1	0.8	0.5	0.3	0.0
Premix ^b^	2.5	2.5	2.5	2.5	2.5
Limestone	6.0	4.5	3.0	1.5	0.0
Dicalcium phosphate	12.5	12.0	11.2	10.5	10.0
Salt	2.0	2.0	2.0	2.0	2.0
Sunflower oil	0.11	0.08	0.06	0.03	0.00
Total	1000.00	1000.00	1000.00	1000.00	1000.00
Calculated values					
Digestible energy (MJ/kg)	13.40	13.38	13.41	13.40	13.39
Crude protein (g/kg)	175.3	175.4	175.4	175.2	175.1
Lysine (g/kg)	9.2	9.2	9.2	9.2	9.2
Ca (g/kg)	8.4	8.5	8.4	8.4	8.5
P (g/kg)	5.9	6.0	5.9	5.9	6.0

^a^ Black Soldier fly larvae meal. ^b^ Supplied the following amounts per kilogram premix (MeloVit 14PG 0.25%): 2,600,000 IU of vitamin A; 700,000 IU of vitamin D3; 20,000 mg of vitamin E as tocopherol acetate; 800 mg of vitamin K3; 800 mg of vitamin B1; 7200 mg of vitamin B3 (niacin); 4000 mg of vitamin 5 (calcium D-pantothenate); 400,000 µg of folic acid; 100,000 µg of biotin; 10,000 µg of vitamin B12; Fe (Fe-II-sulphate) at 35,000 mg; Zn (Zn oxide) at 30,000 mg; Mn (Mn oxide) at 18,000 mg; Cu (Cu-II-sulphate) at 6000 mg; Se (sodium selenite) at 120 mg; and I (calcium iodide) at 100 mg.

**Table 3:** Analysed composition of pig diets used in the experiment (as fed basis) ^a^.

Ingredients/Diets	T0	T30	T60	T90	T120
Gross energy (MJ/kg)	16.84	16.85	17.11	17.07	17.01
Dry matter (g/kg)	897.4	890.9	903.3	894.1	907.2
Crude protein (g/kg)	163.1	166.4	170.8	172.3	167.3
Crude fat (g/kg)	21.2	24.7	29.4	33.6	34.0
Total fibres (g/kg)	39.7	49.0	43.9	36.2	37.7
Acid detergent fibres (g/kg)	59.0	60.5	65.0	53.5	55.0
Acid detergent lignin (g/kg)	27.5	24.0	33.2	19.6	29.5
Calcium (g/kg)	9.13	8.83	8.62	9.19	8.56
Total Phosphorus (g/kg)	7.70	7.44	7.85	6.83	7.67
Indispensable amino acids (g/kg)					
Arginine	12.23	11.19	10.37	8.87	10.06
Histidine	6.17	6.28	7.13	6.55	6.18
Isoleucine	8.09	7.70	7.76	7.11	7.52
Leucine	14.95	14.09	14.30	13.29	13.92
Lysine	11.77	10.99	11.15	9.45	10.56
Methionine	2.63	2.37	2.69	2.55	2.75
Phenylalanine	9.81	9.02	9.12	8.37	8.80
Threonine	7.34	7.00	7.22	6.62	7.22
Tryptophan	3.92	4.41	3.90	4.08	4.88
Valine	9.48	9.29	9.72	9.12	10.05
Dispensable amino acids (g/kg)					
Alanine	8.55	8.58	9.54	8.79	9.98
Aspartic acid	18.17	17.03	17.59	15.34	16.69
Cystine	3.54	2.88	3.17	2.70	2.75
Glutamic acid	40.97	36.79	36.23	33.76	34.03
Proline	13.00	12.27	12.44	12.46	13.00
Tyrosine	7.16	6.99	7.68	6.86	7.82
Serine	9.16	8.89	9.09	8.09	8.71
Glycine	8.15	7.81	8.24	7.76	8.57
Total indispensable amino acids	86.39	82.34	83.36	76.01	81.94
Total dispensable amino acids	108.7	101.24	103.98	95.76	101.55
Total amino acids	195.09	183.58	187.34	171.77	183.49

^a^ Analysed in technical duplicates. T0, T30, T60, T90, T120: diets containing 0, 30, 60, 90, and 120 g/kg of Black Soldier fly larvae meal in replacement of soyabean meal.

**Table 4:** The effect of Black Soldier fly larvae meal inclusion in feed on growth performance, digestible energy, and nutrient digestibility coefficients in growing pigs.

Variables/Diets	T0	T30	T60	T90	T120	SEM	*p*-Value	L	Q	DEV
Start weight (kg/pig)	31.9	32	31.8	31.8	31.8	2.07	1.000	0.949	1.000	0.997
End weight (kg/pig)	60.8	59.5	60.4	58.7	58.9	2.86	0.935	0.481	0.962	0.86
FI (kg/pig/day)	1.89	1.85	1.92	1.87	1.82	0.05	0.278	0.237	0.243	0.303
WG (kg/pig/day)	0.76	0.72	0.75	0.71	0.71	0.042	0.634	0.237	0.929	0.575
FCR (kg:kg)	2.524	2.581	2.576	2.654	2.566	0.1235	0.885	0.579	0.533	0.806
Faecal moisture (kg/kg)	0.763	0.751	0.754	0.747	0.766	0.006	0.134	0.944	0.024	0.383
DE (MJ/kg)	12.95	13.04	12.77	13.31	13.2	0.208	0.406	0.261	0.607	0.294
CPD	0.694	0.684	0.674	0.715	0.708	0.0291	0.619	0.369	0.481	0.521
DMD	0.766	0.763	0.734	0.772	0.772	0.0114	0.123	0.536	0.096	0.119
TFD	0.277	0.392	0.285	0.209	0.161	0.0852	0.101	0.037	0.196	0.384
ADFD	0.092	0.21	0.164	0.128	0.251	0.0525	0.239	0.163	0.918	0.168
FD	0.774 ^a^	0.814 ^ab^	0.814 ^ab^	0.844 ^bc^	0.860 ^c^	0.0143	0.002	< 0.001	0.744	0.519

T0, T30, T60, T90, and T120: diets contain 0, 30, 60, 90, and 120 g/kg of Black Soldier fly larvae meal in replacement of soyabean meal; SEM: pooled standard error of the mean; *p*-value: Fisher’s probability; L: orthogonal polynomial contrast for a linear response; Q: orthogonal polynomial contrast for a quadratic response; DEV (deviation): orthogonal polynomial contrast for deviation from a linear or quadratic response; values in a row with different superscript letters differ significantly; start weight: weight of the pigs at the beginning of the experiment at 119 days of age; end weight: weight of the pigs at the end of the experiment at 157 days of age; FI: daily feed intake; WG: daily weight gain; FCR: feed conversion ratio; faecal moisture: water content of fresh faeces (kg/kg); DE: dietary digestible energy; CPD: total tract crude protein digestibility coefficient; DMD: total tract dry matter digestibility coefficient; TFD: total tract total fibre digestibility coefficient; AIFD: total tract acid detergent fibre digestibility coefficient; FD: total tract fat digestibility coefficient.

**Table 5:** The effect of Black Soldier fly larvae meal inclusion in feed on haematology and the blood biochemistry profile of growing pigs.

Variable/ Diets	T0	T30	T60	T90	T120	SEM	*p*-Value	L	Q	DEV
RBC (×10^12^/L)	7.4	7.3	7.4	7	7.1	0.18	0.58	0.213	0.731	0.561
WBC (×10^9^/L)	15.7 ^a^	14.7 ^a^	14.1 ^a^	15.3 ^a^	18.9 ^b^	0.99	0.016	0.034	0.005	0.832
Plt (K/uL)	503	404	433	504	522	36.6	0.12	0.239	0.052	0.31
Hct (%)	41.0	41.0	41.0	38.0	39.0	1.20	0.258	0.100	0.616	0.309
Hb (g/dL)	12.79	12.74	12.95	11.81	12.06	0.397	0.2	0.067	0.617	0.297
SOD (U/mL)	2.0 ^a^	4.1 ^bc^	4.6 ^c^	3.7 ^b^	4.1 ^bc^	0.19	<0.001	<0.001	<0.001	<0.001
TAC (pg/mL)	117.8 ^a^	140.6 ^b^	152.6 ^b^	153.8 ^b^	151.5 ^b^	5.75	<0.001	<0.001	0.007	0.907
CAT (U/mL)	6.0 ^b^	5.8 ^b^	5.0 ^a^	4.7 ^a^	4.8 ^a^	0.25	0.002	<0.001	0.243	0.389
GPx (U/mL)	64.6 ^c^	47.6 ^a^	52.2 ^b^	52.5 ^b^	52.8 ^b^	1.04	<0.001	<0.001	<0.001	<0.001
IL-1β (ng/mL)	150.9 ^a^	171.6 ^b^	185.4 ^b^	170.2 ^b^	176.0 ^b^	5.6	0.002	0.009	0.008	0.105
IL-6 (pg/mL)	148.1 ^a^	171.8 ^b^	174.0 ^b^	180.2 ^b^	177.5 ^b^	4.84	<0.001	<0.001	<0.011	0.469
IL-10 (pg/mL)	180.4 ^c^	150.9 ^b^	124.6 ^a^	149.0 ^b^	144.6 ^b^	5.04	<0.001	<0.001	<0.001	0.004
INF-γ (pg/mL)	152.6 ^a^	160.5 ^ab^	168.5 ^bc^	169.8 ^bc^	173.8 ^c^	3.6	0.002	<0.001	0.291	0.841
TNF-α (pg/mL)	65.7 ^a^	70.9 ^b^	69.7 ^b^	68.8 ^b^	65.0 ^a^	1.04	<0.001	0.273	<0.001	0.293
ROS (a.u.)	0.2 ^a^	1.7 ^c^	1.0 ^b^	1.6 ^c^	1.0 ^b^	0.07	<0.001	<0.001	<0.001	<0.001
•NO (a.u.)	6.7 ^a^	11.6 ^b^	12.3 ^b^	12.2 ^b^	6.8 ^a^	0.71	<0.001	0.741	<0.001	0.407
•Asc (a.u.)	0.29 ^a^	0.56 ^b^	0.55 ^b^	0.72 ^c^	0.75 ^c^	0.054	<0.001	<0.001	0.182	0.192
MDA (µmol/mL	2.8 ^a^	4.0 ^b^	4.7 ^bc^	4.9 ^bc^	5.2 ^c^	0.34	<0.001	<0.001	0.088	0.827
8-OHdG (ng/mL)	12.3 ^a^	13.8 ^a^	16.0 ^b^	13.9 ^a^	14.0 ^a^	0.62	0.004	0.083	0.004	0.071
5-MSL (a.u)	0.7 ^a^	1.3 ^b^	1.3 ^b^	1.2 ^b^	1.2 ^b^	0.05	<0.001	<0.001	<0.001	<0.001
AGE (mg/mL)	278.0 ^b^	241.0 ^a^	253.0 ^a^	255.0 ^a^	253.0 ^a^	6.92	0.011	0.116	0.028	0.034
PC (nmol/mg)	2.8 ^a^	5.3 ^b^	7.3 ^c^	9.0 ^d^	8.2 ^c^	0.31	<0.001	<0.001	<0.001	0.083
TEMPOL (a.u.)	7.5 ^a^	13.5 ^b^	14.6 ^b^	14.1 ^b^	13.6 ^b^	0.66	<0.001	<0.001	<0.001	0.081

T0, T30, T60, T90, and T120: diets contain 0, 30, 60, 90, and 120 g/kg of Black Soldier fly larvae meal in replacement of soyabean meal; SEM: pooled standard error of the mean; *p*-value: Fisher’s probability; L: orthogonal polynomial contrast for a linear response; Q: orthogonal polynomial contrast for a quadratic response; DEV (deviation): orthogonal polynomial contrast for deviation from a linear or quadratic response; values in a row with different superscript letters differ significantly; RBCs: red blood cells (erythrocytes); WBCs: white blood cells (leukocytes); Plt: platelet; Hct: haematocrit; Hb: haemoglobin; SOD: superoxide dismutase; TAC: total antioxidant capacity; CAT: catalase; GPx: glutathione peroxidase; IL-1β, IL-10, IL-6, ITF-γ, and TNF-α: cytokine concentrations in blood plasma; ROS: reactive oxygen species; •NO: nitric oxide; •Asc: ascorbate radicals; MDA: malondialdehyde; 8-OHdG: 8-hydroxydeoxyguanosine; 5-MSL: protein oxidation; AGEs: advanced glycation end products; PC: protein carbonyl damage; TEMPOL: membrane-permeable piperidine nitroxide.

## Data Availability

The data that support the findings of this study are available upon reasonable request from the corresponding author.
